# The Analysis of ceRNA Networks and Tumor Microenvironment in Endometrial Cancer

**DOI:** 10.7150/jca.93364

**Published:** 2024-02-24

**Authors:** Jian Huang, Xiaoyue Yang, Shen Xu, Ping Li

**Affiliations:** 1 Clinical and Translational Research Center, Shanghai First Maternity and Infant Hospital, Tongji University School of Medicine, Shanghai, China.; 2 The International Peace Maternity and Child Health Hospital, School of Medicine, Shanghai Jiao Tong University, Shanghai 200030, China.; 3 Shanghai Key Laboratory of Embryo Original Diseases, Shanghai 200030, China.; 4 Blood Transfusion Department, Ruijin Hospital, Shanghai Jiao Tong University School of Medicine, Shanghai, China.; 5 Department of Gynecology, Affiliated Hospital of Jiangsu University, Zhenjiang, Jiangsu, China.

**Keywords:** Endometrial carcinoma, ceRNA, Prognosis, Immune cells infiltration, Machine learning

## Abstract

**Background:** Endometrial carcinoma is a life-threatening and aggressive tumor that affects women worldwide. ceRNAs and carcinoma-infiltrating immunocytes can be associated with tumor formation and progression. Therefore, investigating the unique mechanisms underlying endometrial carcinoma is crucial.

**Methods:** Prognostic nomograms were constructed based on the differentially expressed genes between normal and tumor tissues. Twenty types of tumor immune infiltrating cells in uterine corpus endometrial carcinoma (UCEC) were examined using CIBERSORT. To identify the potential signaling pathways, the associations among essential ceRNA network genes and important immunocytes were investigated using the co-expression assay.

**Results:** Differential analysis identified 3636 mRNAs, 249 miRNAs, and 252 lncRNAs unique to UCEC. The ceRNA network was constructed using the interplays between 19 lncRNA-miRNA pairs and 434 miRNA-mRNA pairs. Furthermore, CIBERSORT and ceRNA integration analysis revealed that immune cells, including dendritic cells and natural killer cells, and associated ceRNAs such as LRP8, HDGF, PPARGC1B, and TEAD1 can appropriately predict prognosis. A receiver operating characteristic curve was constructed to predict patient outcomes.

**Conclusions:** Using a nomogram, we predicted the outcomes of patients with UCEC Furthermore, we revealed its significance in improving clinical management.

## Introduction

Endometrial cancer (EC) is clinically heterogeneous and the most common gynecological malignancy worldwide 1. Since 2008, the incidence of EC has increased by 21%, with the fatality rate increasing by >100% in the past two decades 1, 2. As of right now, endometrial cancer ranks second in frequency among gynecological cancers in China 3. In 2015, there was an annual growth rate of 3.7 percent with approximately 69,000 new cases of endometrial cancer detected and 16,000 fatalities 3. Furthermore, the prevalence of EC continues to significantly increase. At present, no clear and effective therapy is available for EC 4. Although surgery can cure several patients with EC, many women with EC have a dismal prognosis 1, 2, 4. Furthermore, owing to their aggressive disposition and a lack of reliable indicators, distant metastasis is common 5. Therefore, investigating the molecular processes and establishing prognostic markers for EC are vital 6.

Although noncoding RNAs (ncRNAs) are frequently used to represent nonprotein-encoding RNAs such as long noncoding RNAs (lncRNAs) and microRNAs (miRNAs), this does not indicate that such RNAs do not have any functions 7-9. For the first time in 2011, Salmena et al. suggested the competitive endogenous RNA (ceRNA) network and demonstrated a novel mechanism underlying the interactions among miRNAs, lncRNAs, and mRNAs. This network is crucial for cancer development and many other biological processes 10. However, the mechanism of the ceRNA network in EC remains unknown 11. Furthermore, there are no studies on ceRNA and immune cells in EC 10, 12.

In the present study, using the expression profile information of The Cancer Genome Atlas uterine corpus endometrial carcinoma (TCGA-UCEC) samples, a UCEC prognosis-associated ceRNA network was constructed. TCGA contains the gene expression profiles of miRNAs, lncRNAs, and mRNAs and the clinical data for UCEC. Bioinformatics methods were used to identify associated differentially expressed genes (DEGs) and construct ceRNA networks. In addition, using the CIBERSORT algorithm, which estimates relative RNA transcript subsets by clarifying cell types, the immunocytes and their percentages in UCEC were elucidated. Lastly, to determine the novel therapeutic approaches and channels for patient treatment and lengthen their survival times, the interaction between immune cells associated with UCEC and ceRNA networks was examined. Immune cells and ceRNA were used to establish nomograms that may predict the prognosis of patients with UCEC. Using this network, the underlying signaling pathways may be used to predict the survival of patients with UCEC.

## Materials and Methods

### Data Collection and Analysis

The relevant UCEC clinical, miRNA sequencing, and RNA sequencing (RNA-seq) data were obtained from TCGA (https://portal.gdc.cancer.gov/). The profiles of 575 samples, including 552 UCEC samples and 23 tumor-free tissue samples, were mapped using fragments per kilobase of exon per million reads and HTseq-count. Furthermore, the demographic and survival data of all patients were collected from TCGA. Patients with insufficient clinical data were excluded from the study. Therefore, 87 individuals without any tumors and 461 individuals with UCEC were enrolled in the present study. The clinicopathological information of the 548 individuals is presented in **Table 1**.

### Analysis of DEGs

Using the edgeR method 13, the differentially expressed miRNAs, mRNAs, and lncRNAs were clarified using the following criteria: logFC (fold change) < -1.0 or > 1.0 and false discovery rate (FDR) p < 0.05 14. To construct heatmaps and volcano plots, the R package ggplot2 was used 15.

### Establishment of a ceRNA Network-Based Prognostic Model

Using TCGA, survival time and status were retrieved using the clinical information of patients with UCEC as the base data. By combining clinical information with ceRNA network node information, the prognostic roles of the node genes were evaluated using Kaplan-Meier (K-M) plots and univariate Cox analysis 16. Next, after eliminating overlapping components using Lasso regression (accomplished using the “glmnet” tool in R), a ceRNA network-based prognostic model was constructed via multivariate Cox regression analysis 16.

### Construction of the ceRNA Regulatory Network

Based on previous statistical analysis, data on the retrieval of lncRNA-miRNA and miRNA-mRNA pairs and interplays were collected from miRTarBase (https://mirtarbase.cuhk.edu.cn/) and PITA (https://genie.weizmann.ac.il/pubs/mir07/mir07_exe.html) 17. Thereafter, Cytoscape 3.7.1 was used to generate the miRNA-mRNA and miRNA-lncRNA interaction pairs 18. The lncRNA-miRNA-mRNA ceRNA regulatory network is based on miRNA-targeting genes and expression 19.

### Survival Assessment and Nomograms in the ceRNA Network

The associations between biomarker levels and prognosis depicted in the ceRNA network and the survival status of patients with UCEC were demonstrated using K-M plots 20. Then, a nomogram based on Lasso regression and univariate and multivariate Cox analyses was developed for the prognostic prediction of patients with UCEC 16. Based on the correlation between biomarker expression and prognosis, the scores for each biomarker can be obtained; these scores can be added to obtain the final score, representing the likelihood of survival for 1, 2, and 3 years 21. To determine the sensitivity and accuracy of the nomogram, the receiver operating characteristic (ROC) using the survival ROC R package and calibration curves were simultaneously used 22.

### CIBERSORT Estimation

Using CIBERSORT, an analytical method, it is possible to determine the diversity and percentages of various cell types within a heterogeneous cell population 23. Furthermore, each cell category and its quantity per sample can be instantly assessed. In addition, the cytological factors for chief biomarkers in the molecular processes of the ceRNA network can be investigated. To estimate the percentage of 22 different immunocyte categories in UCEC, CIBERSORT was used as previously described 11. Data were deemed appropriate for subsequent study only when CIBERSORT p < 0.05.

### Statistical Analysis

R ver. 3.5.1 (Institute for Statistics and Mathematics, Vienna, Austria; www.r-project.org) was used to conduct statistical analyses. Statistical significance was evaluated at a p-value of <0.05.

## Results

### Recognition of the Significant DEGs in UCEC

**Figure 1:**
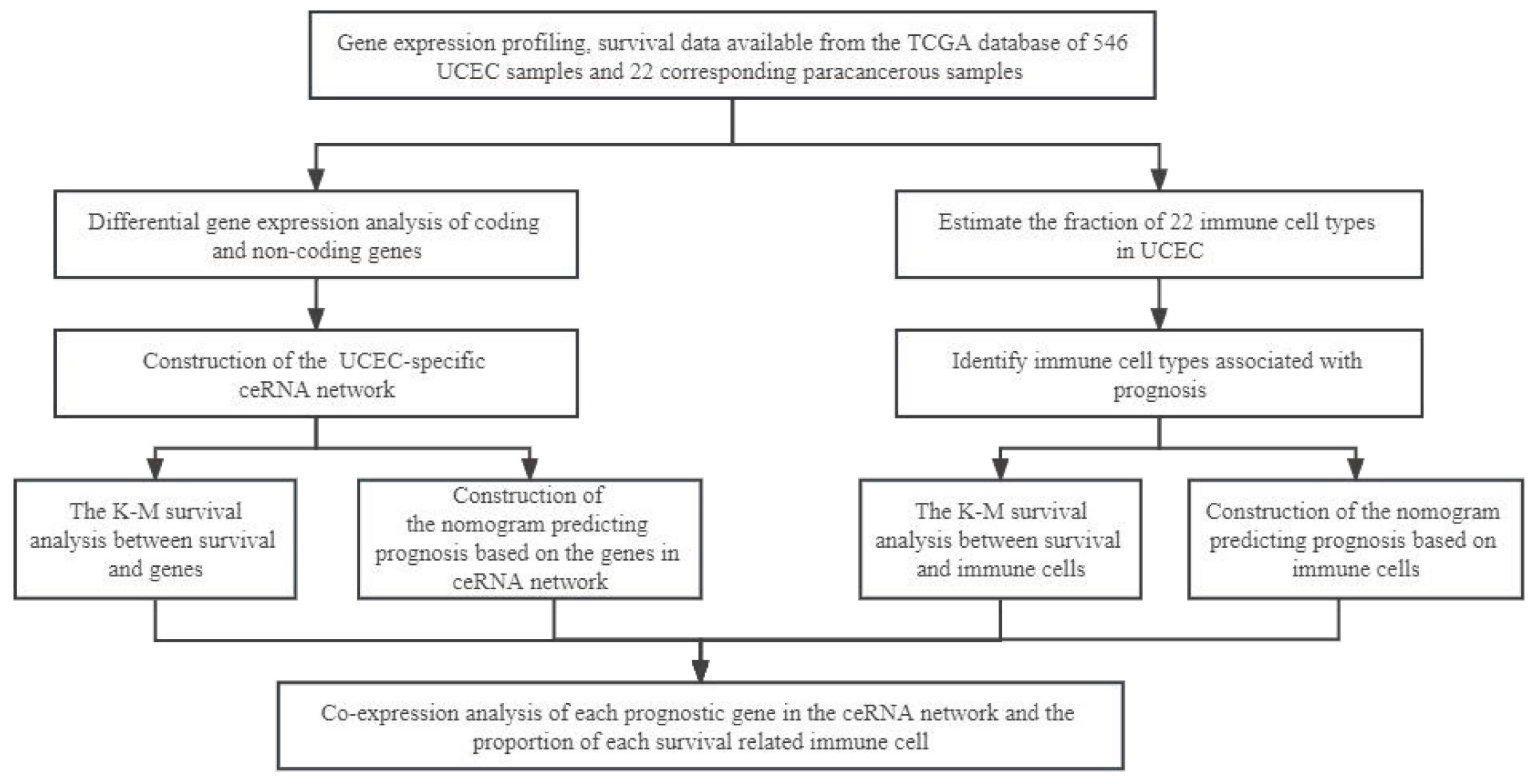
The flow diagram of the whole analytical procedure.

**Figure 2:**
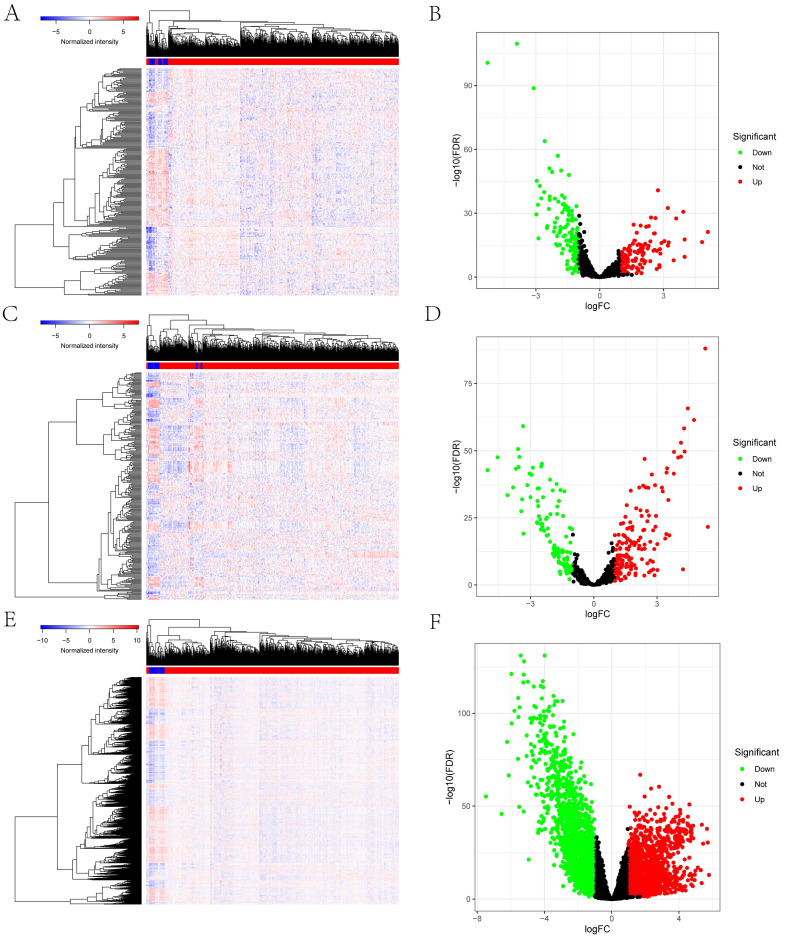
Identification of the differentially expressed mRNAs, lncRNAs, miRNAs between UCEC and normal tissue. The cutoffs which we set was log(fold-change) > 1.0 or < -1.0 and FDR < 0.05.** (A)** The heatmap of genome-wide differentially expressed lncRNAs. **(B)** The volcano plot showed that a total of 122 upregulated lncRNAs and 130 downregulated lncRNAs were screened out. **(C)** The heatmap of genome-wide differentially expressed miRNAs. **(D)** The volcano plot showed that a total of 146 upregulated miRNAs and 103 downregulated miRNAs were screened out. **(E)** The heatmap of genome-wide differentially expressed mRNAs. **(F)** The volcano plot showed that a total of 1690 upregulated mRNAs and 1946 downregulated mRNAs were screened out. Green and red represents downregulated and upregulated mRNAs, respectively.

**Figure 1** schematically describes the methods and findings of the present study. TCGA was used to extract the general characteristics of each patient, which are presented in **Table 1**. We examined the RNA-seq data of 22 paracancerous samples and 546 UCEC samples in TCGA. In total, 252 UCEC-specific lncRNAs (130 downregulated and 122 upregulated) (Figures 2A, B), 249 miRNAs (103 downregulated and 146 upregulated) (Figures 2C, D), and 3636 mRNAs (1946 downregulated and 1690 upregulated) (Figures 2E, F) were identified as differentially expressed RNAs from TCGA-UCEC and normalized using the following thresholds: FDR < 0.05 and log FC < -1.0 or > 1.0.

### ceRNA Network Creation and Survival Assessment in UCEC

**Figure 3:**
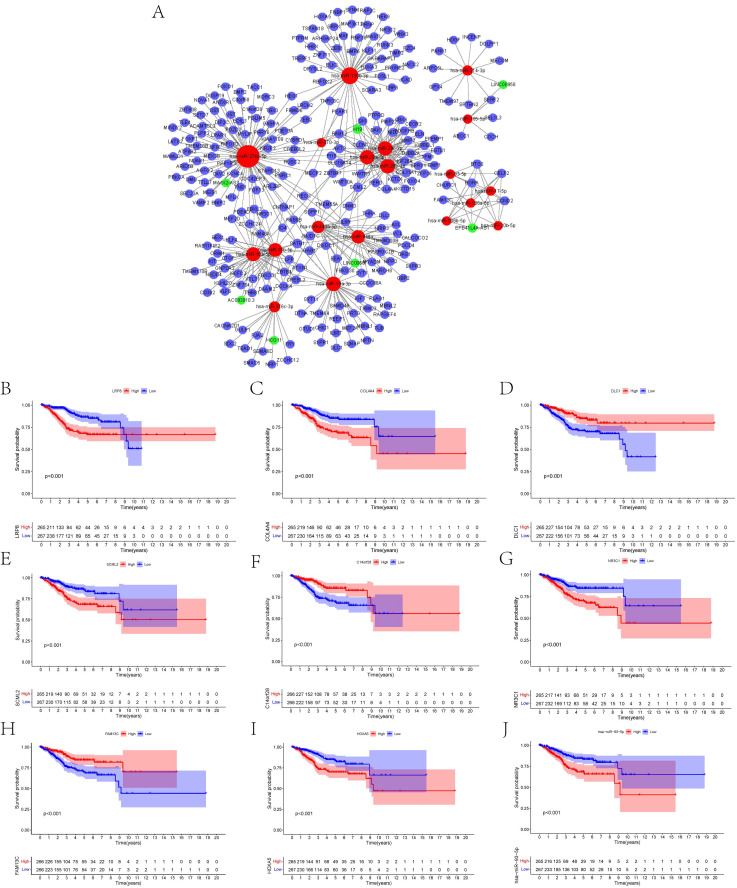
** (A)** Overview of the lncRNA-miRNA-mRNA ceRNA network of UCEC with 19 lncRNA-miRNA couples and 434 miRNA-mRNA pairs. Red balls represent miRNAs, green balls represent lncRNAs, and blue balls represent protein-coding mRNAs. The Kaplan-Meier survival curves based on the expression of biomarkers involved in ceRNA network related to the UCEC shows that **(B)** LRP8 (P < 0.001), **(C)** COL4A4 (P < 0.001), **(D)** DLC1 (P < 0.001), **(E)** SCML2 (P < 0.001), **(F)** C14orf28 (P < 0.001), **(G)** NR3C1 (P < 0.001), **(H)** FAM13C (P < 0.001), **(I)** HOXA5 (P < 0.001) and** (J)** hsa-miR-93-5p (P < 0.001) had significantly prognostic values.

To construct the ceRNA network comprising 247 genes, the interplays between 19 lncRNA-miRNA pairs and 434 miRNA-mRNA pairs were used (**Figure 3A, Supplementary Tables 1, 2**). The associations between the biomarkers in the UCEC-based ceRNA network and prognosis were determined using K-M plots, the log-rank test, and Cox regression analysis. K-M analysis of the ceRNA network revealed the top 10 significant genes, including LRP8 (p = 2.68E-05), COL4A4 (p = 6.75E-05), DLC1 (p = 0.000260844), SCML2 (p = 0.000313436), C14orf28 (p = 0.00035164), NR3C1 (p = 0.000351683), FAM13C (p = 0.0004065), and HOXA5 (p = 0.000492153), and hsa-miR-93-5p (P = 0.00049857) (**Figures 3B-J**). **Supplementary Table 3** lists all the significant genes**.**

### Establishment and Evaluation of the ceRNA Network-Based Prognostic Model

**Figure 4:**
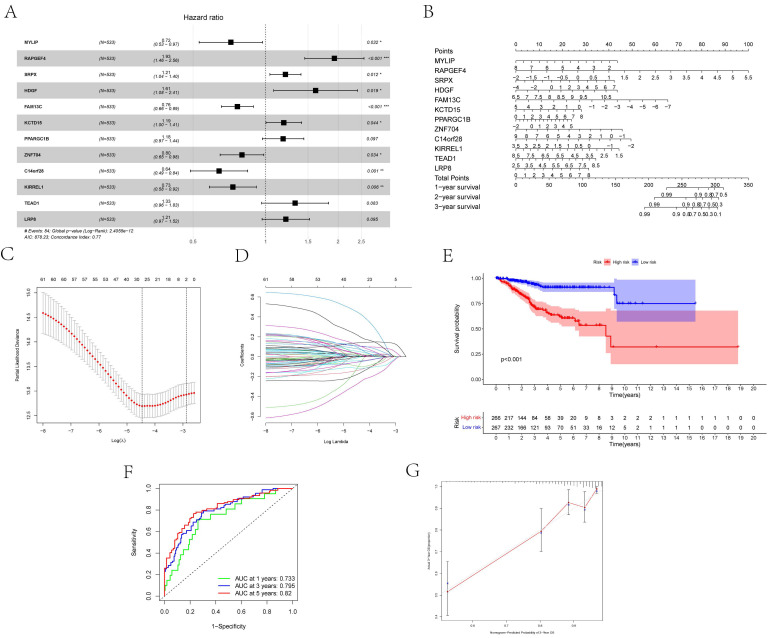
** (A)** The results of the Cox proportional hazards model based on the multivariate Cox regression. **(B)** The nomogram for predicting outcome of patients based on ceRNAs. **(C, D)** The model selected by Lasso regression. **(E)** The result of the Kaplan-Meier curves suggested risk score had prognostic value for UCEC patients (P < 0.001). **(F, G)** The ROC curves and the calibration indicated the acceptable accuracy of the nomogram [Area Under Curve (AUC) of 1-year survival: 0.733, AUC of 3-year survival: 0.795; AUC of 5-year survival: 0.82].

**Figure 5:**
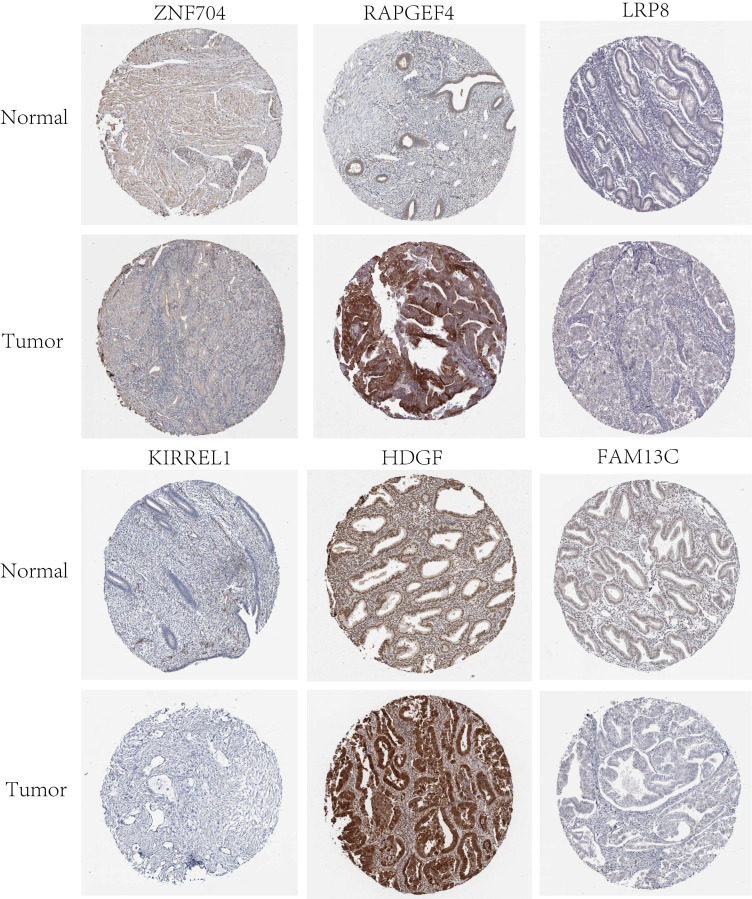
Validation of protein expression patterns dictated by select genes in both normal and UCEC tissues was performed utilizing samples procured from the Human Protein Atlas (HPA) database.

Twelve relevant prognostic biomarkers were identified as essential elements for the ceRNA network; they were used to construct a novel multivariate model (**Figure 4A**). Using this model, the nomogram was depicted (**Figure 4B**). Using Lasso regression and univariate Cox regression analysis, the pivotal network genes were identified. Multivariate Cox regression analysis identified 12 important genes for the model (**Figures 4C, D**). The K-M plot revealed a substantial difference in the high-risk and low-risk populations. Simultaneously, K-M curves revealed that the survival time of the low-risk population was prominently longer than that of the high-risk population (**Figure 4E**). Furthermore, ROC and calibration curves (**Figures 4F, G**) revealed that the nomogram exhibited adequate accuracy and discrimination, with AUC values of 0.733, 0.795, and 0.82 for 1-, 3-, and 5-year survival rates, respectively. Occasionally, we determined the regulation of the proteins encoded by some of these genes. **Figure 5** demonstrates that RAPGEF4, LRP8, and heparin-binding growth factor (HDGF) were positively expressed in EC tissues compared with nontumor tissues. In contrast, ZNF704, KIRREL1, and FAM13C were positively expressed in normal endometrial tissues.

### Composition of Carcinoma-Infiltrating Immunocytes in UCEC

**Figure 6:**
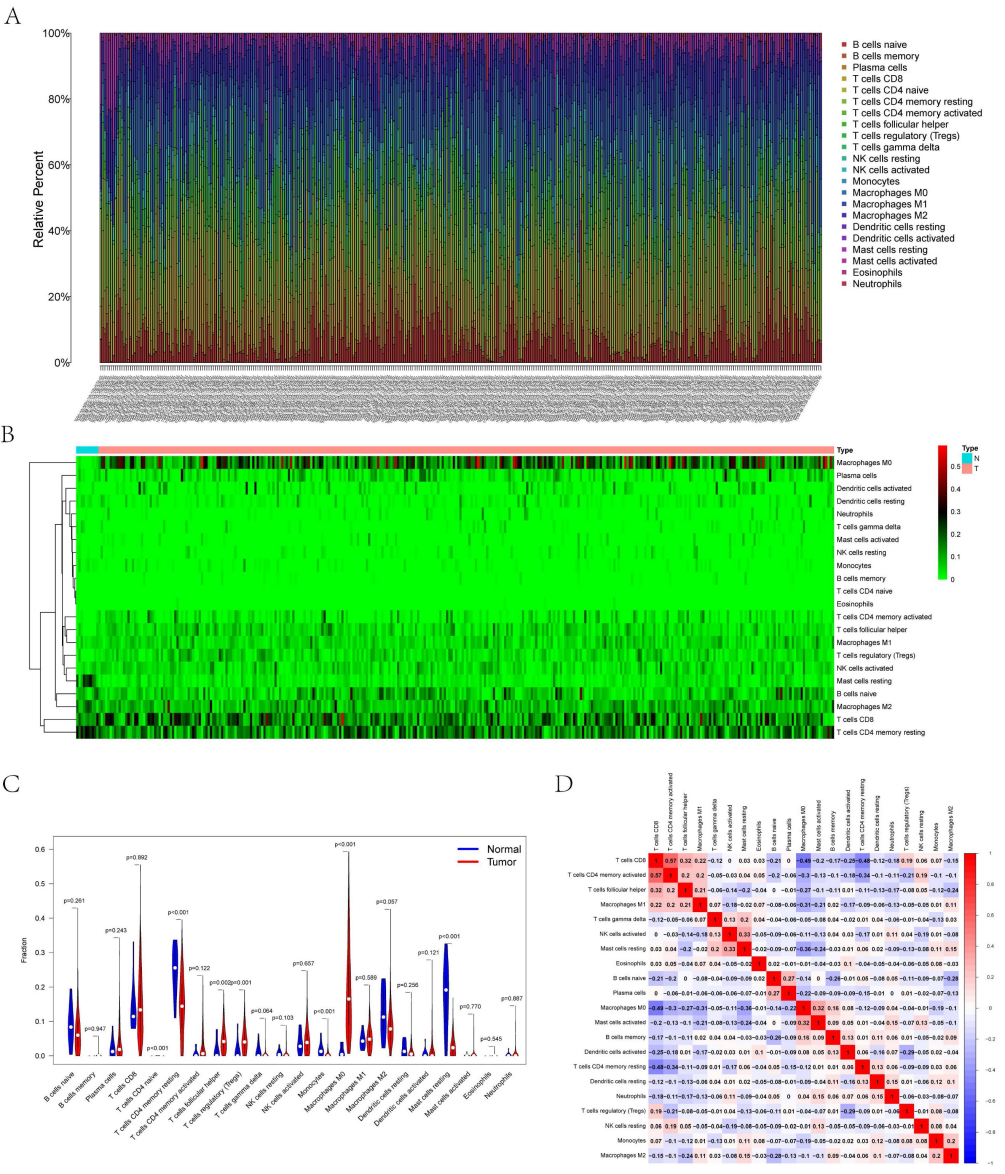
** (A)** Bar plot showing the composition of immune cells and relative percent in UCEC. Different colors represent different cell types. **(B)** Heatmap of tumor-infiltrating cells in tumor tissues estimated by CIBERSORT algorithm in UCEC. Annotations on top show clustering of samples. **(C)** The violin plot showed the proportion of cells between normal tissue and tumor tissue. The blue and red bars represent the tumor group and primary tumor group, respectively. **(D)** The result of the co-expression analysis (Pearson analysis) between significant tumor-infiltrating immune cells.

**Figures 6A and B** illustrate the histograms and thermograms displaying the composition of 22 types of CIBERSORT-identified immunocytes in UCEC. The goal of compositional estimation was to investigate the differences in the tumor microenvironments (TMEs) of healthy and carcinoma samples. The Wilcoxon rank-sum outcomes, as depicted in the violin plot, revealed a significantly higher percentage of memory CD4^+^ T cells and mast cells in the bone metastatic melanoma group than in the normal group (p < 0.001) and a comparatively higher percentage of M0 macrophages in the tumor group (p < 0.001) (**Figure 6C**). Pearson's correlation analysis was used to perform the co-expression assay among the proportions of carcinoma-infiltrating immunocytes (**Figure 6D**). The assay revealed a significantly positive association between CD8 T cells and memory CD4^+^ T cells (r = 0.57); however, they were significantly and negatively associated with M1 macrophages (r = -0.49).

### Clinical Relevance of the Immune Cells

**Figure 7:**
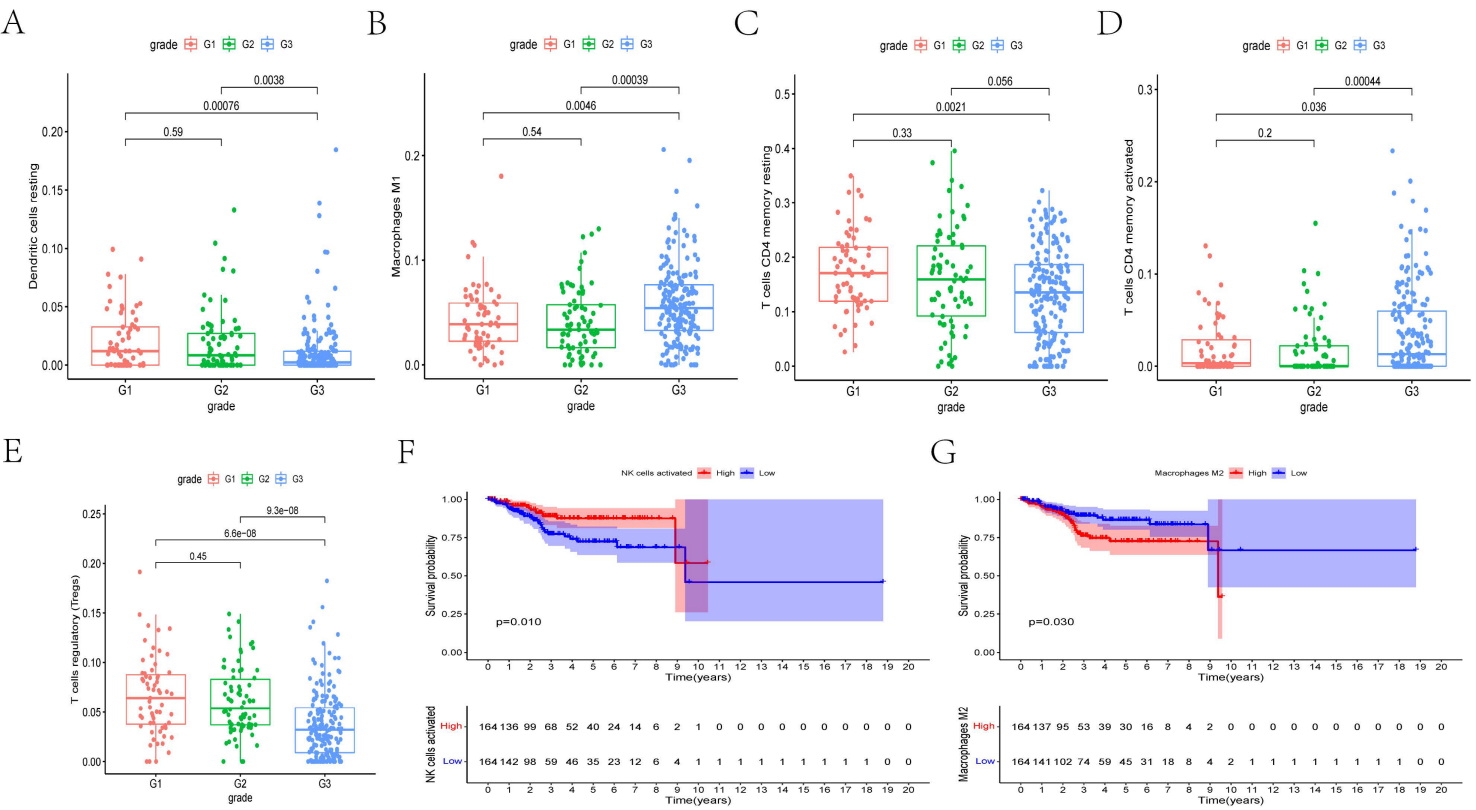
** (A-E)** The box plots showed the fraction of Dendritic cells resting, Macrophages M1, T cells CD4 memory resting, T cells CD4 memory activated, and T cells regulatory (Tregs) between different grades of cancer. **(E, F)** The Kaplan-Meier survival curves of NK cells activated and Macrophages M2.

We determined whether there is an association between the percentages of various immunocyte subtypes and prognosis using K-M plots and nonparameter testing. The co-expression assay between immunocytes and clinical prognosis revealed that the fractions of M1 macrophages, resting dendritic cells, regulatory T cells (Tregs), and memory CD4^+^ T cells significantly differed among different cancer grades (**Figures 7A-E**). Furthermore, the fractions of activated natural killer cells (P = 0.010) and M2 macrophages (p = 0.030) were significantly associated with overall survival (**Figures 7F, G**).

### Analysis of Immune Cells for Prognosis

**Figure 8:**
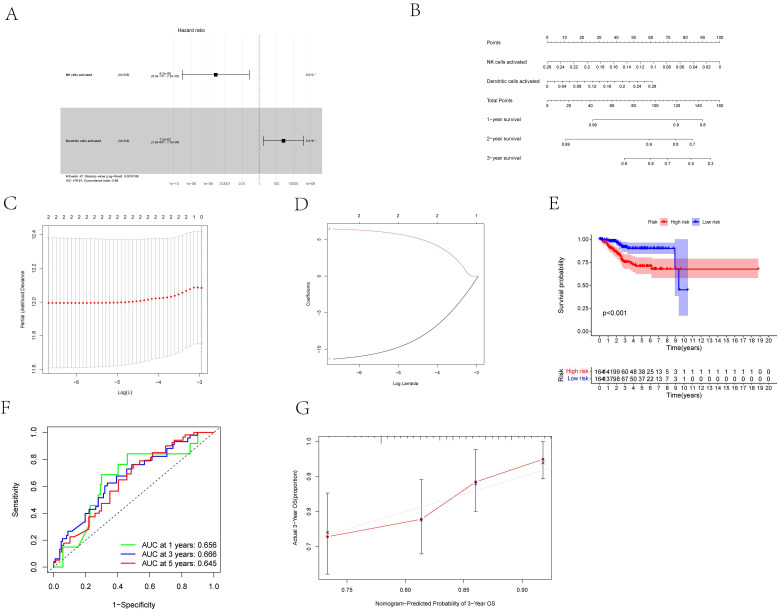
**(A)** The Cox proportional hazards model was integrated by different types of immune cells by the multivariate Cox regression. **(B)** The nomogram was based on prognosis-related immune cells for predicting outcome of the UCEC patients. **(C, D)** The model selected by Lasso regression. **(E)** The result of the Kaplan-Meier curves suggested risk score based on prognosis-related immune cells had prognostic value for UCEC patients (P < 0.001).** (F)** The ROC was calculated on account of the significant immune cells that were associated with survival [AUC of 1-year survival: 0.656, AUC of 3-year survival: 0.666; AUC of 5-year survival: 0.645]. **(G)** The calibration curves were used to evaluate the accuracy of the nomogram.

First, we considered two possible prognosis-associated biomarkers as pivotal members among the 22 immunocyte subtypes and constructed a novel multivariate model (**Figure 8A**). Using this model, two types of critical tumor immunocytes related to 1-, 2- and 3-year overall survival probabilities were depicted as gene models in the form of a nomogram (**Figure 8B**). To examine the efficacy of these genes for modeling, Lasso regression analysis was conducted (**Figures 8C, D**). **Figure 8E** illustrates the immunocyte percentages and survival status of each group. Furthermore, calibration and ROC curves revealed the discrimination and consistency of the nomogram, with AUC values of 0.656, 0.666, and 0.645 for 1-, 3-, and 5-year survival rates, respectively (**Figures 8F, G**).

### Co-expression Assay between Immunocytes and Genes

**Figure 9:**
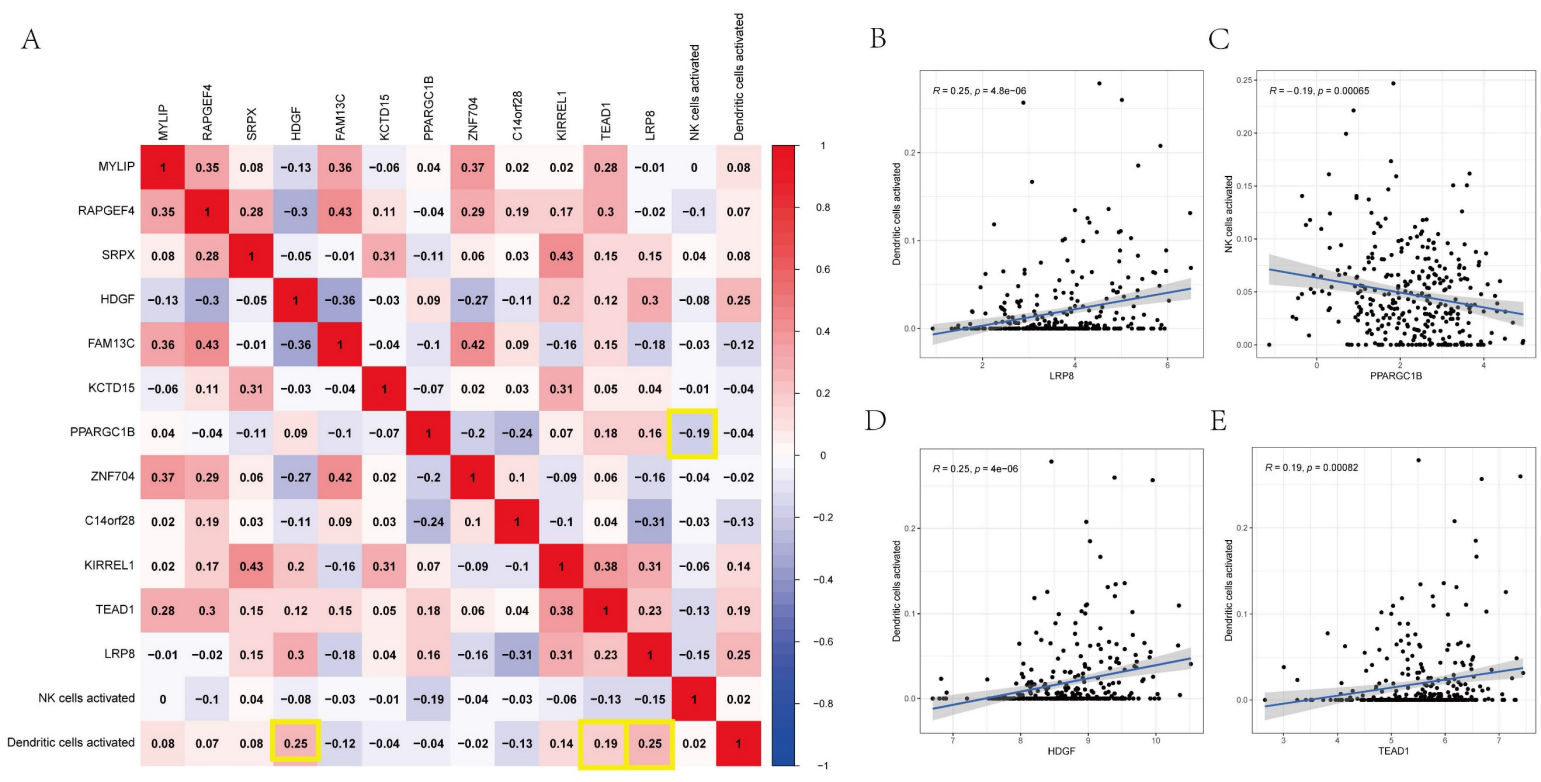
** (A)** The co-expression heatmap revealed the fractions of tumor-infiltrating immune cells and the key components in the ceRNA network. **(B-E)** Scatterplots further illustrate the exact relationship between Dendritic cells activated and LRP8 (R = 0.25, P = 4.8e-06), HDGF (R = 0.25, P = 4e-06) and TEAD1 (R = 0.19, P = 0.00082), NK cells activated and PPARGC1B (R = -0.19, P = 0.00065). These could be used to describe the important relationship between the key biomarkers.

Using Pearson's correlation analysis, **Figure 9A** depicts some significant patterns of co-expression between crucial ceRNA network members and pivotal immunocyte members. The activated dendritic cell fraction was positively associated with the expression of LRP8 (r = 0.25, p = 4.8e-06), HDGF (r = 0.25, p = 4e-06), and TEAD1 (r = 0.19, p = 0.00082). However, the activated natural killer cell fraction was negatively associated with PPARGC1B (r = -0.19, p = 0.00065) (**Figures 9B-E**).

## Discussion

EC is a common type of gynecological carcinoma 5, 24. In America, it is the fourth most common carcinoma among women, after breast, lung, and colorectal cancers 5, 24. A ceRNA network comprises lncRNAs and miRNAs, which belong to protein-coding mRNAs 8. Many studies have reported the critical role of miRNAs in the regulation of carcinoma-associated genes 10. miRNAs exert a regulatory role on mRNA function by primarily integrating with MREs, resulting in mRNA degradation 10. lncRNAs are transcripts that are >200 nucleotides long and have no or limited protein-coding potential; they can be used to diagnose and treat UCEC 25-27. Therefore, the lncRNAs and carcinoma-infiltrating immunocytes that are differentially expressed in UCEC, which has largely been ignored in previous studies, piqued our interest. It is significantly vital to clarify the pathogenesis of UCEC and identify innovative carcinoma biomarkers.

In the present study, we focused on TME-infiltrating immunocytes and the ceRNA network. Using TCGA-UCEC, 252 UCEC-specific lncRNAs, 249 miRNAs, and 3636 mRNAs were identified as differentially expressed RNAs. By fusing the miRNA interplays with mRNAs or lncRNAs, a UCEC-based ceRNA network was established, encompassing 19 lncRNA-miRNA pairs and 434 miRNA-mRNA pairs. CIBERSORT was used to identify the surrounding carcinoma-infiltrating immunocytes between the normal and UCEC groups. Then, using the chosen ceRNAs and surrounding tumor immunocytes, prognostic nomograms were constructed. Using the significant TME-infiltrating immunocytes and mRNAs, two risk models were constructed. In addition, the prognosis of UCEC was predicted based on the AUCs of the two nomograms, with values of 0.656, 0.666, and 0.645 for 1-, 3-, and 5-year survival rates, respectively. Furthermore, we observed that immunocytes such as natural killer and dendritic cells and the related ceRNAs of LRP8, PPARGC1B, HDGF, and TEAD1 can accurately predict prognosis. Finally, correlation analysis revealed dramatic correlations between dendritic cells and LRP8 (r = 0.25, p = 4.8e-06), HDGF (r = 0.25, p = 4e-06), and TEAD1 (r = 0.19, p = 0.00082). However, dendritic cells were negatively associated with PPARGC1B (r = -0.370, p < 0.001). We suggest that these ceRNAs and their respective relevant mechanisms play critical roles in the prediction and treatment of UCEC.

LRP8 has already emerged as a promising biomarker for the diagnosis and management of carcinomas as well as for predicting their prognosis 28. Some studies have revealed that higher LRP8 expression is associated with poor patient survival 29. In addition, a Chinese population-based study has revealed that the single-nucleotide variants of PPARγ, PPARGC1A, and PPARGC1B may be related to the susceptibility factors of gastric cancer in an eastern Chinese population 30. HDGF plays vital roles in the generation of blood vessels and mitosis and facilitates malignant processes such as cellular multiplication, invasion, and migration 31-34. Some studies have demonstrated the Hippo-TEAD pathway modulates cell proliferation and function in both nonmalignant mature differentiated and malignant cells 35. A preliminary investigation has revealed that TEAD1 binds to the NGF promotor and that YAP1/TEAD1 increases its transcription, resulting in improved cell invasion 36. However, only few studies have investigated these ceRNAs.

The TME plays pivotal roles in the growth and progression of carcinoma cells. Immune cells in the TME have either tumor-opposing or tumor-promoting effects 11. Depending on their intracancer functions, carcinoma-associated immunocytes in the TME can be classified into two subtypes: tumor-promoting immunocytes (TPICs) and tumor-antagonistic immunocytes (TAICs) 23. TAICs primarily comprise M1 macrophages, N1 neutrophils, dendritic cells, natural killer cells, and effector T cells 37. On the other hand, the TPIC subtype comprises Tregs and suppressor cells of myeloid origin. Carcinoma-associated immunocytes improve tumor development via cytokine release and metastasis, which are achieved by generating matrix-degrading enzymes and growth factors 38. Furthermore, immune infiltration can affect clinical prognosis 37.

During tumor development and progression, LRP8, PPARGC1B, HDGF, and TEAD1 can kill them quickly if several nearby cells display oncogenic transformation-associated surface markers 39, 40. Natural killer cells are unique immune cells; their ability to boost T cell responses suggests that they can be used as anticancer agents 39-42. As distinct antigen-presenting cells, dendritic cells play a fundamental role in the development and modulation of both adaptive and innate immune responses 43-45. Steinman, who received a Nobel Prize in Biomedicine in 2011, discovered that dendritic cells are the commander of the human immune cell system, commanding and leading various immune system functions 46. Dendritic cells are responsible for antigen phagocytosis, processing, and presentation and familiarizing helper T and B cells with the characteristics of cancer cells that have been fought 47, 48. After being informed by dendritic cells, helper T cells activate cytotoxic T cells, macrophages, natural killer cells, and B cells that have been stimulated by dendritic cells. After recognizing cancer cells, cytotoxic T cells kill them 49. Some cytotoxic T cells are converted to a memory phenotype via helper T cells. This will make it easier for macrophages to decompose the cancer cells they engulf; on the other hand, natural killer cells can directly attack cancer cells. Therefore, we suggest that LRP8, PPARGC1B, HDGF, and TEAD1, as ceRNAs, are associated with the active participation of natural killer and dendritic cells in UCEC formation. The ceRNAs enlisted in our prognostic panel can be potential innovative targets for managing UCEC. Nevertheless, additional studies are warranted for further exploring the mechanisms.

Although we established an UCEC-specific ceRNA network and filtration for possible prognostic biomarkers, the present study still has some limitations. The sample size should be increased to verify our findings, and more samples are warranted for corroborating the prediction capability of the prognostic models. In addition, because our study included a mere multidimensional correlation investigation, further experiments are warranted to comprehensively determine the functions of hub genes to verify the potential biological mechanisms of these ceRNAs in UCEC.

## Conclusions

In the present study, we constructed a UCEC-associated ceRNA network using bioinformatics methods. We found that UCEC may be related to LRP8, HDGF, PPARGC1B, and TEAD1 as well as its role in regulating dendritic and natural killer cells by using ceRNA networks and infiltrating immunocytes. Nomograms were constructed using these networks and immunocytes to predict the survival status of the UCEC population. Using AUC values, we demonstrated that the nomograms have great practicability. This nomogram-based integrative analysis can help improve the personalized management of patients with UCEC. Lastly, we hypothesized that the mechanism of ceRNAs is valuable for UCEC prognosis, whereas infiltrating immunocytes play significant roles in UCEC development.

## Supplementary Material

Supplementary tables.

## Figures and Tables

**Table 1:** Patient information of Uterine Corpus Endometrial Carcinoma cohort.

Clinical features	TCGA-UECE(N=548)	
	No	%
**OS**		
0	461	84.12
1	87	15.88
**Age**		
<=60	209	38.35
>60	336	61.65
**Stage**		
I	339	62.20
II	52	9.54
III	124	22.75
IV	30	5.50
**Grade**		
I	99	18.07
II	122	22.26
III	316	57.66
IV	11	2.01

## Abbreviations

EC: Endometrial cancer
DEGs: differential expression genes
CIBERSORT: cell type identification by estimating relative subsets of RNA transcripts
ncRNAs: Non-coding RNAs
miRNAs: including microRNAs
lncRNAs: long non-coding RNAs
ceRNA: competitive endogenous RNA
TCGA: The Cancer Genome Atlas
FDR: false discovery rate
FC: fold change
K-M: Kaplan-Meier
ROC: receiver operating characteristic
