# Supplementary material for: The Analysis of ceRNA Networks and Tumor Microenvironment in Endometrial Cancer
Source: J Cancer. 2024 Feb 24;15(8):2147–59. doi: 10.7150/jca.93364 (PMC10937290; doi:10.7150/jca.93364)
Supplement: Supplementary file 1 — Supplementary tables. [file jcav15p2147s1.pdf]

Supplementary Table 1 | The PPI nodes of DEGs involved in the ceRNA network of UCEC.

symbol	type	num-Interactions
FBXL3	pc	3
MYLIP	pc	1
NFIX	pc	1
MAMLD1	pc	1
IGF1	pc	1
WWTR1	pc	3
SAMD4A	pc	1
RUNX3	pc	1
BAK1	pc	3
VCL	pc	2
TIMP2	pc	1
CELF2	pc	5
PER3	pc	3
ELN	pc	3
MYLK	pc	1
KIF26A	pc	3
KLF6	pc	2
SYT1	pc	3
MEF2A	pc	1
REEP1	pc	1
RORA	pc	5
NEDD4	pc	2
CYBRD1	pc	1
FRY	pc	1
NUAK1	pc	1
KLHL20	pc	2
TNRC6C	pc	2
TNS1	pc	1
COL4A4	pc	3
MECOM	pc	1
MMP2	pc	3
NID2	pc	3
RAPGEF4	pc	1
SEL1L3	pc	1
DPYSL2	pc	1
TMEM38B	pc	1
NRP1	pc	1
CECR2	pc	3
RBFOX2	pc	1
COCH	pc	1

SEC23A	pc	1
SRPX	pc	1
SCML2	pc	7
EEA1	pc	3
ARL2BP	pc	1
ABCC1	pc	1
FBXO31	pc	2
XYLT1	pc	2
CAV2	pc	1
HOXA3	pc	1
HOXA5	pc	1
AGFG2	pc	1
PRUNE2	pc	1
TLE4	pc	1
RAB11FIP2	pc	2
CNTNAP1	pc	3
MPP2	pc	2
TMEM97	pc	1
PMP22	pc	3
GAB1	pc	4
ELK3	pc	1
CREBL2	pc	3
HCFC2	pc	2
RIPOR2	pc	1
UST	pc	1
QKI	pc	5
PDE10A	pc	1
FAM46A	pc	2
PDE4D	pc	3
NR3C1	pc	3
TRIM23	pc	1
SMAD5	pc	1
PDGFRB	pc	3
ZBTB47	pc	6
FOXP1	pc	1
CCDC88A	pc	2
EFEMP1	pc	2
KCNC4	pc	1
MEF2D	pc	3
AKT3	pc	4
FBXO30	pc	2
CTGF	pc	2
CCND2	pc	5
NEK9	pc	1

PPP1R3C	pc	1
MASTL	pc	1
RECK	pc	2
RAP2C	pc	1
TRERF1	pc	1
ATXN1	pc	3
BMP2	pc	1
THRA	pc	2
SGPP1	pc	3
ZFP36	pc	3
DOCK4	pc	3
MINDY2	pc	1
FGF13	pc	1
LRCH2	pc	2
H19	lnc	5
SYT11	pc	1
STARD13	pc	1
BEX1	pc	2
PDZD2	pc	1
IL6ST	pc	1
KCTD1	pc	3
EMP1	pc	3
DTNA	pc	1
TBX3	pc	1
WNT10A	pc	2
CALCOCO2	pc	1
KLF4	pc	2
ARPC5L	pc	1
RNF38	pc	1
TUBB2A	pc	3
PI15	pc	4
THBS1	pc	2
SEMA6D	pc	1
TTLL7	pc	1
ARHGAP24	pc	1
KIAA1109	pc	1
GABARAPL1	pc	1
MAP3K12	pc	1
MBNL2	pc	1
NOVA1	pc	1
FRMD6	pc	2
SERF2	pc	2
BNIP2	pc	1
TPM1	pc	3

HDGF	pc	1
ATP8B2	pc	1
MEIS1	pc	1
GULP1	pc	1
LPP	pc	3
SETD7	pc	1
DAAM2	pc	2
PNRC1	pc	1
ZNF711	pc	1
TACC1	pc	1
NACC2	pc	1
ZEB1	pc	1
FAM13C	pc	2
INCENP	pc	1
ARID5B	pc	1
LATS2	pc	1
PDCD4	pc	1
DIXDC1	pc	2
FOXO1	pc	1
CRIM1	pc	2
DIP2C	pc	1
NR3C2	pc	1
DST	pc	1
MBNL1	pc	1
PANK1	pc	1
HHEX	pc	1
BMP6	pc	1
PTPRD	pc	4
KCTD15	pc	3
CACNA2D1	pc	1
PRKCA	pc	1
GABPA	pc	1
TMEM55A	pc	3
PPARGC1B	pc	2
ADAMTSL3	pc	1
NPTN	pc	1
LRP8	pc	1
KIT	pc	2
TSPAN18	pc	1
MORC3	pc	1
BTG2	pc	5
IL6R	pc	2
PLPP3	pc	1
GBP2	pc	1

DUSP19	pc	1
SLC16A14	pc	4
PDLIM5	pc	1
CDC42EP3	pc	1
GNPDA2	pc	2
CLDN1	pc	3
FSTL1	pc	3
SLMAP	pc	1
SPRY1	pc	3
EDIL3	pc	1
ZNF704	pc	2
DLC1	pc	1
OTUD1	pc	1
MARCH8	pc	2
ZCCHC24	pc	3
JCAD	pc	1
RAB8B	pc	2
TUB	pc	1
PRTG	pc	1
DOLPP1	pc	1
GPX4	pc	1
AXL	pc	2
SCARA3	pc	1
MECP2	pc	3
SDC2	pc	1
ZEB2	pc	2
ANTXR1	pc	1
S1PR1	pc	1
PRNP	pc	1
JMJD1C	pc	4
KLF11	pc	1
ID4	pc	2
PTPRM	pc	1
HEG1	pc	2
SPTBN2	pc	1
ZBTB4	pc	3
ZCCHC12	pc	1
FZD4	pc	1
NRIP3	pc	2
FOSL1	pc	1
ZBTB38	pc	1
MAF	pc	1
C14orf28	pc	1
MYADM	pc	2

GAS1	pc	2
TMEM64	pc	1
PLAG1	pc	1
C3orf58	pc	1
CREB3L2	pc	1
SYNM	pc	1
SATB1	pc	7
MEX3B	pc	1
CBX6	pc	3
KIRREL1	pc	3
FOXO4	pc	3
PRR16	pc	1
NR2F2	pc	1
TEAD1	pc	1
FNBP1	pc	1
PEAR1	pc	1
WNK3	pc	1
DNM3	pc	4
LPAR1	pc	1
DLL1	pc	2
RUSC2	pc	1
DMD	pc	1
PJA2	pc	1
CHIC1	pc	1
COL15A1	pc	3
TMEM170B	pc	1
RAB12	pc	5
S1PR3	pc	1
VAMP2	pc	1
EPB41L4A-AS1	lnc	5
HCG11	lnc	1
MAGI2-AS3	lnc	1
CD302	pc	1
LINC00958	lnc	2
TMEM200B	pc	2
CHURC1	pc	2
AC093010.3	lnc	2
LINC00667	lnc	3
hsa-miR-106a-5p	mir	6
hsa-miR-106b-5p	mir	5
hsa-miR-130b-3p	mir	49
hsa-miR-17-5p	mir	6
hsa-miR-185-5p	mir	5
hsa-miR-18a-5p	mir	33

hsa-miR-18b-5p	mir	31
hsa-miR-19a-3p	mir	36
hsa-miR-20b-5p	mir	6
hsa-miR-214-3p	mir	11
hsa-miR-29a-3p	mir	34
hsa-miR-29b-3p	mir	35
hsa-miR-29c-3p	mir	35
hsa-miR-370-3p	mir	5
hsa-miR-374b-5p	mir	83
hsa-miR-376c-3p	mir	15
hsa-miR-449a	mir	29
hsa-miR-449b-5p	mir	23
hsa-miR-93-5p	mir	6

Supplementary Table 2 | The lncRNA–miRNA–mRNA ceRNA network of UCEC.

fromNode	toNode	Network
MAGI2-AS3	hsa-miR-374b-5p	ceRNA
AC093010.3	hsa-miR-18a-5p	ceRNA
AC093010.3	hsa-miR-18b-5p	ceRNA
LINC00667	hsa-miR-449a	ceRNA
LINC00667	hsa-miR-449b-5p	ceRNA
LINC00667	hsa-miR-19a-3p	ceRNA
HCG11	hsa-miR-376c-3p	ceRNA
LINC00958	hsa-miR-185-5p	ceRNA
LINC00958	hsa-miR-214-3p	ceRNA
EPB41L4A-AS1	hsa-miR-17-5p	ceRNA
EPB41L4A-AS1	hsa-miR-20b-5p	ceRNA
EPB41L4A-AS1	hsa-miR-93-5p	ceRNA
EPB41L4A-AS1	hsa-miR-106b-5p	ceRNA
EPB41L4A-AS1	hsa-miR-106a-5p	ceRNA
H19	hsa-miR-29c-3p	ceRNA
H19	hsa-miR-29b-3p	ceRNA
H19	hsa-miR-29a-3p	ceRNA
H19	hsa-miR-130b-3p	ceRNA
H19	hsa-miR-370-3p	ceRNA
AKT3	hsa-miR-374b-5p	ceRNA
ZCCHC24	hsa-miR-374b-5p	ceRNA
RECK	hsa-miR-374b-5p	ceRNA
PI15	hsa-miR-374b-5p	ceRNA
RIPOR2	hsa-miR-374b-5p	ceRNA
PPP1R3C	hsa-miR-374b-5p	ceRNA
TACC1	hsa-miR-374b-5p	ceRNA
ZEB2	hsa-miR-374b-5p	ceRNA
MYLK	hsa-miR-374b-5p	ceRNA
DMD	hsa-miR-374b-5p	ceRNA
UST	hsa-miR-374b-5p	ceRNA
NR3C1	hsa-miR-374b-5p	ceRNA
TNS1	hsa-miR-374b-5p	ceRNA
C14orf28	hsa-miR-374b-5p	ceRNA
LRCH2	hsa-miR-374b-5p	ceRNA
RAB8B	hsa-miR-374b-5p	ceRNA
FBXL3	hsa-miR-374b-5p	ceRNA
CYBRD1	hsa-miR-374b-5p	ceRNA
FRMD6	hsa-miR-374b-5p	ceRNA
TMEM55A	hsa-miR-374b-5p	ceRNA
CAV2	hsa-miR-374b-5p	ceRNA
ZBTB38	hsa-miR-374b-5p	ceRNA

NOVA1	hsa-miR-374b-5p	ceRNA
TLE4	hsa-miR-374b-5p	ceRNA
MECP2	hsa-miR-374b-5p	ceRNA
RUSC2	hsa-miR-374b-5p	ceRNA
STARD13	hsa-miR-374b-5p	ceRNA
CDC42EP3	hsa-miR-374b-5p	ceRNA
PDE10A	hsa-miR-374b-5p	ceRNA
SETD7	hsa-miR-374b-5p	ceRNA
MINDY2	hsa-miR-374b-5p	ceRNA
NR2F2	hsa-miR-374b-5p	ceRNA
TBX3	hsa-miR-374b-5p	ceRNA
MAMLD1	hsa-miR-374b-5p	ceRNA
EDIL3	hsa-miR-374b-5p	ceRNA
VAMP2	hsa-miR-374b-5p	ceRNA
MORC3	hsa-miR-374b-5p	ceRNA
RAB12	hsa-miR-374b-5p	ceRNA
PNRC1	hsa-miR-374b-5p	ceRNA
FOXO1	hsa-miR-374b-5p	ceRNA
ADAMTSL3	hsa-miR-374b-5p	ceRNA
LATS2	hsa-miR-374b-5p	ceRNA
ATP8B2	hsa-miR-374b-5p	ceRNA
FGF13	hsa-miR-374b-5p	ceRNA
BNIP2	hsa-miR-374b-5p	ceRNA
HEG1	hsa-miR-374b-5p	ceRNA
PRKCA	hsa-miR-374b-5p	ceRNA
SATB1	hsa-miR-374b-5p	ceRNA
MEF2D	hsa-miR-374b-5p	ceRNA
AGFG2	hsa-miR-374b-5p	ceRNA
GABPA	hsa-miR-374b-5p	ceRNA
CREB3L2	hsa-miR-374b-5p	ceRNA
FOXP1	hsa-miR-374b-5p	ceRNA
KIAA1109	hsa-miR-374b-5p	ceRNA
SEC23A	hsa-miR-374b-5p	ceRNA
PRR16	hsa-miR-374b-5p	ceRNA
PDZD2	hsa-miR-374b-5p	ceRNA
NUAK1	hsa-miR-374b-5p	ceRNA
PDE4D	hsa-miR-374b-5p	ceRNA
DIP2C	hsa-miR-374b-5p	ceRNA
ANTXR1	hsa-miR-374b-5p	ceRNA
JMJD1C	hsa-miR-374b-5p	ceRNA
TMEM38B	hsa-miR-374b-5p	ceRNA
KCNC4	hsa-miR-374b-5p	ceRNA
ARID5B	hsa-miR-374b-5p	ceRNA
SGPP1	hsa-miR-374b-5p	ceRNA

MYLIP	hsa-miR-374b-5p	ceRNA
PLPP3	hsa-miR-374b-5p	ceRNA
ARL2BP	hsa-miR-374b-5p	ceRNA
FAM46A	hsa-miR-374b-5p	ceRNA
DUSP19	hsa-miR-374b-5p	ceRNA
DST	hsa-miR-374b-5p	ceRNA
PDLIM5	hsa-miR-374b-5p	ceRNA
SLC16A14	hsa-miR-374b-5p	ceRNA
ID4	hsa-miR-374b-5p	ceRNA
LPAR1	hsa-miR-374b-5p	ceRNA
C3orf58	hsa-miR-374b-5p	ceRNA
MEX3B	hsa-miR-374b-5p	ceRNA
TTLL7	hsa-miR-374b-5p	ceRNA
NFIX	hsa-miR-374b-5p	ceRNA
BMP2	hsa-miR-374b-5p	ceRNA
MEIS1	hsa-miR-374b-5p	ceRNA
ZCCHC24	hsa-miR-18a-5p	ceRNA
ZCCHC24	hsa-miR-18b-5p	ceRNA
HCFC2	hsa-miR-18a-5p	ceRNA
HCFC2	hsa-miR-18b-5p	ceRNA
CD302	hsa-miR-18a-5p	ceRNA
NR3C1	hsa-miR-18a-5p	ceRNA
NR3C1	hsa-miR-18b-5p	ceRNA
NEDD4	hsa-miR-18a-5p	ceRNA
NEDD4	hsa-miR-18b-5p	ceRNA
FBXL3	hsa-miR-18a-5p	ceRNA
FBXL3	hsa-miR-18b-5p	ceRNA
ZBTB4	hsa-miR-18a-5p	ceRNA
ZBTB4	hsa-miR-18b-5p	ceRNA
LPP	hsa-miR-18a-5p	ceRNA
LPP	hsa-miR-18b-5p	ceRNA
XYLT1	hsa-miR-18a-5p	ceRNA
XYLT1	hsa-miR-18b-5p	ceRNA
DOCK4	hsa-miR-18a-5p	ceRNA
DOCK4	hsa-miR-18b-5p	ceRNA
GAB1	hsa-miR-18a-5p	ceRNA
GAB1	hsa-miR-18b-5p	ceRNA
KLF4	hsa-miR-18a-5p	ceRNA
KLF4	hsa-miR-18b-5p	ceRNA
CNTNAP1	hsa-miR-18a-5p	ceRNA
CNTNAP1	hsa-miR-18b-5p	ceRNA
FBXO31	hsa-miR-18a-5p	ceRNA
FBXO31	hsa-miR-18b-5p	ceRNA
CREBL2	hsa-miR-18a-5p	ceRNA

CREBL2	hsa-miR-18b-5p	ceRNA
CRIM1	hsa-miR-18a-5p	ceRNA
CRIM1	hsa-miR-18b-5p	ceRNA
ZBTB47	hsa-miR-18a-5p	ceRNA
ZBTB47	hsa-miR-18b-5p	ceRNA
SATB1	hsa-miR-18a-5p	ceRNA
SATB1	hsa-miR-18b-5p	ceRNA
MEF2D	hsa-miR-18a-5p	ceRNA
MEF2D	hsa-miR-18b-5p	ceRNA
RAB11FIP2	hsa-miR-18a-5p	ceRNA
RAB11FIP2	hsa-miR-18b-5p	ceRNA
KLHL20	hsa-miR-18a-5p	ceRNA
KLHL20	hsa-miR-18b-5p	ceRNA
KLF6	hsa-miR-18a-5p	ceRNA
KLF6	hsa-miR-18b-5p	ceRNA
DAAM2	hsa-miR-18a-5p	ceRNA
DAAM2	hsa-miR-18b-5p	ceRNA
THBS1	hsa-miR-18a-5p	ceRNA
THBS1	hsa-miR-18b-5p	ceRNA
ATXN1	hsa-miR-18a-5p	ceRNA
ATXN1	hsa-miR-18b-5p	ceRNA
GNPDA2	hsa-miR-18a-5p	ceRNA
GNPDA2	hsa-miR-18b-5p	ceRNA
CTGF	hsa-miR-18a-5p	ceRNA
CTGF	hsa-miR-18b-5p	ceRNA
PDE4D	hsa-miR-18a-5p	ceRNA
PDE4D	hsa-miR-18b-5p	ceRNA
TMEM170B	hsa-miR-18a-5p	ceRNA
KIT	hsa-miR-18a-5p	ceRNA
KIT	hsa-miR-18b-5p	ceRNA
ZNF704	hsa-miR-18a-5p	ceRNA
ZNF704	hsa-miR-18b-5p	ceRNA
BEX1	hsa-miR-18a-5p	ceRNA
BEX1	hsa-miR-18b-5p	ceRNA
DIXDC1	hsa-miR-449a	ceRNA
DIXDC1	hsa-miR-449b-5p	ceRNA
TMEM200B	hsa-miR-449a	ceRNA
TMEM200B	hsa-miR-449b-5p	ceRNA
RECK	hsa-miR-449a	ceRNA
GAS1	hsa-miR-449a	ceRNA
GAS1	hsa-miR-449b-5p	ceRNA
TUB	hsa-miR-19a-3p	ceRNA
RAPGEF4	hsa-miR-19a-3p	ceRNA
RAB8B	hsa-miR-19a-3p	ceRNA

TRIM23	hsa-miR-19a-3p	ceRNA
TMEM55A	hsa-miR-449a	ceRNA
TMEM55A	hsa-miR-449b-5p	ceRNA
ZBTB4	hsa-miR-19a-3p	ceRNA
LPP	hsa-miR-19a-3p	ceRNA
SLMAP	hsa-miR-19a-3p	ceRNA
REEP1	hsa-miR-19a-3p	ceRNA
SAMD4A	hsa-miR-19a-3p	ceRNA
MECP2	hsa-miR-19a-3p	ceRNA
DOCK4	hsa-miR-19a-3p	ceRNA
S1PR1	hsa-miR-19a-3p	ceRNA
GAB1	hsa-miR-449a	ceRNA
GAB1	hsa-miR-449b-5p	ceRNA
DLC1	hsa-miR-19a-3p	ceRNA
CALCOCO2	hsa-miR-449a	ceRNA
AXL	hsa-miR-449a	ceRNA
AXL	hsa-miR-449b-5p	ceRNA
MBNL2	hsa-miR-19a-3p	ceRNA
MBNL1	hsa-miR-19a-3p	ceRNA
OTUD1	hsa-miR-19a-3p	ceRNA
CREBL2	hsa-miR-19a-3p	ceRNA
MEF2A	hsa-miR-19a-3p	ceRNA
THRA	hsa-miR-449a	ceRNA
THRA	hsa-miR-449b-5p	ceRNA
S1PR3	hsa-miR-449a	ceRNA
MYADM	hsa-miR-449a	ceRNA
MYADM	hsa-miR-449b-5p	ceRNA
MARCH8	hsa-miR-449a	ceRNA
MARCH8	hsa-miR-449b-5p	ceRNA
EEA1	hsa-miR-19a-3p	ceRNA
EEA1	hsa-miR-449a	ceRNA
EEA1	hsa-miR-449b-5p	ceRNA
FBXO30	hsa-miR-449a	ceRNA
FBXO30	hsa-miR-449b-5p	ceRNA
IL6ST	hsa-miR-19a-3p	ceRNA
DTNA	hsa-miR-19a-3p	ceRNA
SATB1	hsa-miR-19a-3p	ceRNA
SATB1	hsa-miR-449a	ceRNA
SATB1	hsa-miR-449b-5p	ceRNA
TMEM64	hsa-miR-19a-3p	ceRNA
PRTG	hsa-miR-19a-3p	ceRNA
NPTN	hsa-miR-19a-3p	ceRNA
SYT1	hsa-miR-19a-3p	ceRNA
SYT1	hsa-miR-449a	ceRNA

SYT1	hsa-miR-449b-5p	ceRNA
VCL	hsa-miR-449a	ceRNA
VCL	hsa-miR-449b-5p	ceRNA
DLL1	hsa-miR-449a	ceRNA
DLL1	hsa-miR-449b-5p	ceRNA
ATXN1	hsa-miR-19a-3p	ceRNA
PDCD4	hsa-miR-449a	ceRNA
JMJD1C	hsa-miR-449a	ceRNA
JMJD1C	hsa-miR-449b-5p	ceRNA
CHIC1	hsa-miR-19a-3p	ceRNA
SGPP1	hsa-miR-449a	ceRNA
SGPP1	hsa-miR-449b-5p	ceRNA
GBP2	hsa-miR-449a	ceRNA
IL6R	hsa-miR-449a	ceRNA
IL6R	hsa-miR-449b-5p	ceRNA
PPARGC1B	hsa-miR-449a	ceRNA
PPARGC1B	hsa-miR-449b-5p	ceRNA
ID4	hsa-miR-19a-3p	ceRNA
SYT11	hsa-miR-19a-3p	ceRNA
PLAG1	hsa-miR-19a-3p	ceRNA
CCDC88A	hsa-miR-19a-3p	ceRNA
CCDC88A	hsa-miR-449a	ceRNA
SCML2	hsa-miR-19a-3p	ceRNA
SCML2	hsa-miR-449a	ceRNA
SCML2	hsa-miR-449b-5p	ceRNA
NRIP3	hsa-miR-449a	ceRNA
NRIP3	hsa-miR-449b-5p	ceRNA
IGF1	hsa-miR-19a-3p	ceRNA
MPP2	hsa-miR-449a	ceRNA
MPP2	hsa-miR-449b-5p	ceRNA
WNT10A	hsa-miR-19a-3p	ceRNA
ZCCHC12	hsa-miR-376c-3p	ceRNA
CACNA2D1	hsa-miR-376c-3p	ceRNA
FRY	hsa-miR-376c-3p	ceRNA
PJA2	hsa-miR-376c-3p	ceRNA
SATB1	hsa-miR-376c-3p	ceRNA
SMAD5	hsa-miR-376c-3p	ceRNA
TEAD1	hsa-miR-376c-3p	ceRNA
GULP1	hsa-miR-376c-3p	ceRNA
JMJD1C	hsa-miR-376c-3p	ceRNA
SEMA6D	hsa-miR-376c-3p	ceRNA
DNM3	hsa-miR-376c-3p	ceRNA
FAM46A	hsa-miR-376c-3p	ceRNA
NRP1	hsa-miR-376c-3p	ceRNA

SDC2	hsa-miR-376c-3p	ceRNA
SEL1L3	hsa-miR-185-5p	ceRNA
ARPC5L	hsa-miR-214-3p	ceRNA
ABCC1	hsa-miR-185-5p	ceRNA
HDGF	hsa-miR-214-3p	ceRNA
SPTBN2	hsa-miR-214-3p	ceRNA
DOLPP1	hsa-miR-214-3p	ceRNA
TMEM97	hsa-miR-214-3p	ceRNA
INCENP	hsa-miR-214-3p	ceRNA
SERF2	hsa-miR-214-3p	ceRNA
SERF2	hsa-miR-185-5p	ceRNA
GPX4	hsa-miR-214-3p	ceRNA
MECOM	hsa-miR-214-3p	ceRNA
PANK1	hsa-miR-214-3p	ceRNA
COCH	hsa-miR-185-5p	ceRNA
FAM13C	hsa-miR-17-5p	ceRNA
FAM13C	hsa-miR-20b-5p	ceRNA
CCND2	hsa-miR-17-5p	ceRNA
CCND2	hsa-miR-93-5p	ceRNA
CCND2	hsa-miR-106b-5p	ceRNA
CCND2	hsa-miR-20b-5p	ceRNA
CCND2	hsa-miR-106a-5p	ceRNA
RORA	hsa-miR-17-5p	ceRNA
RORA	hsa-miR-93-5p	ceRNA
RORA	hsa-miR-106b-5p	ceRNA
RORA	hsa-miR-20b-5p	ceRNA
RORA	hsa-miR-106a-5p	ceRNA
CELF2	hsa-miR-17-5p	ceRNA
CELF2	hsa-miR-93-5p	ceRNA
CELF2	hsa-miR-106b-5p	ceRNA
CELF2	hsa-miR-20b-5p	ceRNA
CELF2	hsa-miR-106a-5p	ceRNA
CHURC1	hsa-miR-93-5p	ceRNA
CHURC1	hsa-miR-106a-5p	ceRNA
BTG2	hsa-miR-17-5p	ceRNA
BTG2	hsa-miR-93-5p	ceRNA
BTG2	hsa-miR-106b-5p	ceRNA
BTG2	hsa-miR-20b-5p	ceRNA
BTG2	hsa-miR-106a-5p	ceRNA
AKT3	hsa-miR-29c-3p	ceRNA
AKT3	hsa-miR-29b-3p	ceRNA
AKT3	hsa-miR-29a-3p	ceRNA
JCAD	hsa-miR-130b-3p	ceRNA
SYNM	hsa-miR-130b-3p	ceRNA

PRUNE2	hsa-miR-130b-3p	ceRNA
PI15	hsa-miR-29c-3p	ceRNA
PI15	hsa-miR-29b-3p	ceRNA
PI15	hsa-miR-29a-3p	ceRNA
SRPX	hsa-miR-130b-3p	ceRNA
ZEB1	hsa-miR-130b-3p	ceRNA
TSPAN18	hsa-miR-130b-3p	ceRNA
ZEB2	hsa-miR-130b-3p	ceRNA
MAF	hsa-miR-130b-3p	ceRNA
PEAR1	hsa-miR-370-3p	ceRNA
LRCH2	hsa-miR-130b-3p	ceRNA
KIF26A	hsa-miR-29c-3p	ceRNA
KIF26A	hsa-miR-29b-3p	ceRNA
KIF26A	hsa-miR-29a-3p	ceRNA
FZD4	hsa-miR-130b-3p	ceRNA
FRMD6	hsa-miR-130b-3p	ceRNA
EFEMP1	hsa-miR-29c-3p	ceRNA
EFEMP1	hsa-miR-29b-3p	ceRNA
PMP22	hsa-miR-29c-3p	ceRNA
PMP22	hsa-miR-29b-3p	ceRNA
PMP22	hsa-miR-29a-3p	ceRNA
FOXO4	hsa-miR-29c-3p	ceRNA
FOXO4	hsa-miR-29b-3p	ceRNA
FOXO4	hsa-miR-29a-3p	ceRNA
TPM1	hsa-miR-29c-3p	ceRNA
TPM1	hsa-miR-29b-3p	ceRNA
TPM1	hsa-miR-29a-3p	ceRNA
MECP2	hsa-miR-130b-3p	ceRNA
NR3C2	hsa-miR-130b-3p	ceRNA
PER3	hsa-miR-29c-3p	ceRNA
PER3	hsa-miR-29b-3p	ceRNA
PER3	hsa-miR-29a-3p	ceRNA
RBFOX2	hsa-miR-130b-3p	ceRNA
RNF38	hsa-miR-130b-3p	ceRNA
CNTNAP1	hsa-miR-370-3p	ceRNA
RAB12	hsa-miR-29c-3p	ceRNA
RAB12	hsa-miR-29b-3p	ceRNA
RAB12	hsa-miR-130b-3p	ceRNA
RAB12	hsa-miR-29a-3p	ceRNA
ZBTB47	hsa-miR-29c-3p	ceRNA
ZBTB47	hsa-miR-29b-3p	ceRNA
ZBTB47	hsa-miR-130b-3p	ceRNA
ZBTB47	hsa-miR-29a-3p	ceRNA
PRNP	hsa-miR-130b-3p	ceRNA

KLF11	hsa-miR-130b-3p	ceRNA
TIMP2	hsa-miR-130b-3p	ceRNA
RAP2C	hsa-miR-130b-3p	ceRNA
HEG1	hsa-miR-130b-3p	ceRNA
ELK3	hsa-miR-130b-3p	ceRNA
DPYSL2	hsa-miR-130b-3p	ceRNA
TRERF1	hsa-miR-130b-3p	ceRNA
MAP3K12	hsa-miR-130b-3p	ceRNA
NACC2	hsa-miR-130b-3p	ceRNA
CBX6	hsa-miR-29c-3p	ceRNA
CBX6	hsa-miR-29b-3p	ceRNA
CBX6	hsa-miR-29a-3p	ceRNA
QKI	hsa-miR-29c-3p	ceRNA
QKI	hsa-miR-29b-3p	ceRNA
QKI	hsa-miR-370-3p	ceRNA
QKI	hsa-miR-130b-3p	ceRNA
QKI	hsa-miR-29a-3p	ceRNA
NEK9	hsa-miR-130b-3p	ceRNA
HHEX	hsa-miR-130b-3p	ceRNA
PDGFRB	hsa-miR-29c-3p	ceRNA
PDGFRB	hsa-miR-29b-3p	ceRNA
PDGFRB	hsa-miR-29a-3p	ceRNA
PTPRD	hsa-miR-29c-3p	ceRNA
PTPRD	hsa-miR-29b-3p	ceRNA
PTPRD	hsa-miR-130b-3p	ceRNA
PTPRD	hsa-miR-29a-3p	ceRNA
FNBP1	hsa-miR-130b-3p	ceRNA
COL15A1	hsa-miR-29c-3p	ceRNA
COL15A1	hsa-miR-29b-3p	ceRNA
COL15A1	hsa-miR-29a-3p	ceRNA
ARHGAP24	hsa-miR-130b-3p	ceRNA
CECR2	hsa-miR-29c-3p	ceRNA
CECR2	hsa-miR-29b-3p	ceRNA
CECR2	hsa-miR-29a-3p	ceRNA
KCTD15	hsa-miR-29c-3p	ceRNA
KCTD15	hsa-miR-29b-3p	ceRNA
KCTD15	hsa-miR-29a-3p	ceRNA
TNRC6C	hsa-miR-370-3p	ceRNA
TNRC6C	hsa-miR-130b-3p	ceRNA
BAK1	hsa-miR-29c-3p	ceRNA
BAK1	hsa-miR-29b-3p	ceRNA
BAK1	hsa-miR-29a-3p	ceRNA
SCARA3	hsa-miR-130b-3p	ceRNA
GABARAPL1	hsa-miR-130b-3p	ceRNA

ZFP36	hsa-miR-29c-3p	ceRNA
ZFP36	hsa-miR-29b-3p	ceRNA
ZFP36	hsa-miR-29a-3p	ceRNA
DNM3	hsa-miR-29c-3p	ceRNA
DNM3	hsa-miR-29b-3p	ceRNA
DNM3	hsa-miR-29a-3p	ceRNA
WWTR1	hsa-miR-29c-3p	ceRNA
WWTR1	hsa-miR-29b-3p	ceRNA
WWTR1	hsa-miR-29a-3p	ceRNA
MASTL	hsa-miR-130b-3p	ceRNA
KIRREL1	hsa-miR-29c-3p	ceRNA
KIRREL1	hsa-miR-29b-3p	ceRNA
KIRREL1	hsa-miR-29a-3p	ceRNA
NID2	hsa-miR-29c-3p	ceRNA
NID2	hsa-miR-29b-3p	ceRNA
NID2	hsa-miR-29a-3p	ceRNA
SLC16A14	hsa-miR-29c-3p	ceRNA
SLC16A14	hsa-miR-29b-3p	ceRNA
SLC16A14	hsa-miR-29a-3p	ceRNA
BMP6	hsa-miR-130b-3p	ceRNA
KCTD1	hsa-miR-29c-3p	ceRNA
KCTD1	hsa-miR-29b-3p	ceRNA
KCTD1	hsa-miR-29a-3p	ceRNA
TUBB2A	hsa-miR-29c-3p	ceRNA
TUBB2A	hsa-miR-29b-3p	ceRNA
TUBB2A	hsa-miR-29a-3p	ceRNA
SPRY1	hsa-miR-29c-3p	ceRNA
SPRY1	hsa-miR-29b-3p	ceRNA
SPRY1	hsa-miR-29a-3p	ceRNA
COL4A4	hsa-miR-29c-3p	ceRNA
COL4A4	hsa-miR-29b-3p	ceRNA
COL4A4	hsa-miR-29a-3p	ceRNA
SCML2	hsa-miR-29c-3p	ceRNA
SCML2	hsa-miR-29b-3p	ceRNA
SCML2	hsa-miR-130b-3p	ceRNA
SCML2	hsa-miR-29a-3p	ceRNA
FSTL1	hsa-miR-29c-3p	ceRNA
FSTL1	hsa-miR-29b-3p	ceRNA
FSTL1	hsa-miR-29a-3p	ceRNA
LRP8	hsa-miR-130b-3p	ceRNA
HOXA5	hsa-miR-130b-3p	ceRNA
ZNF711	hsa-miR-130b-3p	ceRNA
HOXA3	hsa-miR-130b-3p	ceRNA
PTPRM	hsa-miR-130b-3p	ceRNA

ELN	hsa-miR-29c-3p	ceRNA
ELN	hsa-miR-29b-3p	ceRNA
ELN	hsa-miR-29a-3p	ceRNA
EMP1	hsa-miR-29c-3p	ceRNA
EMP1	hsa-miR-29b-3p	ceRNA
EMP1	hsa-miR-29a-3p	ceRNA
CLDN1	hsa-miR-29c-3p	ceRNA
CLDN1	hsa-miR-29b-3p	ceRNA
CLDN1	hsa-miR-29a-3p	ceRNA
RUNX3	hsa-miR-130b-3p	ceRNA
FOSL1	hsa-miR-130b-3p	ceRNA
WNT10A	hsa-miR-130b-3p	ceRNA
WNK3	hsa-miR-130b-3p	ceRNA
MMP2	hsa-miR-29c-3p	ceRNA
MMP2	hsa-miR-29b-3p	ceRNA
MMP2	hsa-miR-29a-3p	ceRNA

Supplementary Table 3 | All the significant genes of the K-M analysis.

genes	p-Value
MYLIP	0.0153007290934359
IGF1	0.0124337113210056
BAK1	0.0022569133989182
KIF26A	0.0495573689471963
NUAK1	0.0160461479039756
COL4A4	6.74631060509912e-05
RAPGEF4	0.0292102165138484
DPYSL2	0.032092309509903
CECR2	0.0326968990517328
SRPX	0.00626369965751139
SCML2	0.00031343648199722
HOXA3	0.0188399616816074
HOXA5	0.000492153373971682
MPP2	0.00210466434097412
TMEM97	0.0327991115544132
UST	0.00136511614703405
NR3C1	0.000351682926748031
CCDC88A	0.0402231528700057
MASTL	0.0106815832159883
RECK	0.0448245774723092
TRERF1	0.0400313855135688
ATXN1	0.00461759994270772
SYT11	0.0427531766036588
PDZD2	0.00191474720814122
IL6ST	0.0362176588774661
DTNA	0.00136154249413334
TTLL7	0.0453691919483391
MAP3K12	0.0300999560919283
SERF2	0.0487934398399135
BNIP2	0.00497981249579815
HDGF	0.0101103838047254
ZNF711	0.0491826176355179
FAM13C	0.000406499805908012
PDCD4	0.0270176311648658
TMEM55A	0.0136527142457998
PPARGC1B	0.0447362756342101
LRP8	2.68435394147337e-05
SLC16A14	0.000653063938187204
CLDN1	0.00135456450269633
ZNF704	0.0357100812350255
DLC1	0.000260843725310544

OTUD1	0.0296050202752319
DOLPP1	0.00512218846546952
GPX4	0.0434400858471713
MECP2	0.043400116212685
PRNP	0.0037372625979184
KLF11	0.021928073460709
ID4	0.00150326591631178
C14orf28	0.000351640004148557
PLAG1	0.00846404176819926
CBX6	0.0342843119638246
KIRREL1	0.00120852999733345
TEAD1	0.014987171817747
WNK3	0.0268541599353778
LPAR1	0.00396818850773717
RUSC2	0.00498378504422636
LINC00958	0.0184133610588973
hsa-miR-17-5p	0.0470199510028908
hsa-miR-18a-5p	0.000616965145500803
hsa-miR-449a	0.00464426858895273
hsa-miR-449b-5p	0.0257353823876809
hsa-miR-93-5p	0.000498570009719423
